# Supplementary material for: Contrasting influences of biogeophysical and biogeochemical impacts of historical land use on global economic inequality
Source: Nat Commun. 2022 May 5;13:2479. doi: 10.1038/s41467-022-30145-6 (PMC9072699; doi:10.1038/s41467-022-30145-6)
Supplement: Supplementary file 1 — Supplementary Information [file 41467_2022_30145_MOESM1_ESM.pdf]

Supplementary Information for

Contrasting Influences of Biogeophysical and Biogeochemical Impacts of Historical Land Use on Global Economic Inequality

Shu Liu[1], Yong Wang[1*], Guang J. Zhang[2], Linyi Wei[1], Bin Wang[1,3,4], Le Yu[1]

[1] Department of Earth System Science, Ministry of Education Key Laboratory for Earth System Modeling, Institute for Global Change Studies, Tsinghua University, Beijing 100084, China.

[2] Scripps Institution of Oceanography, La Jolla, CA, USA.

[3] State Key Laboratory of Numerical Modeling for Atmospheric Sciences and Geophysical Fluid Dynamics, Institute of Atmospheric Physics, Chinese Academy of Sciences, Beijing, China.

[4] College of Earth and Planetary Sciences, University of Chinese Academy of Sciences, Beijing, China.

*Corresponding author. Email: yongw@mail.tsinghua.edu.cn

Supplementary Information includes:

Figs. S1 to S10

Tables S1 to S4

Supplementary References

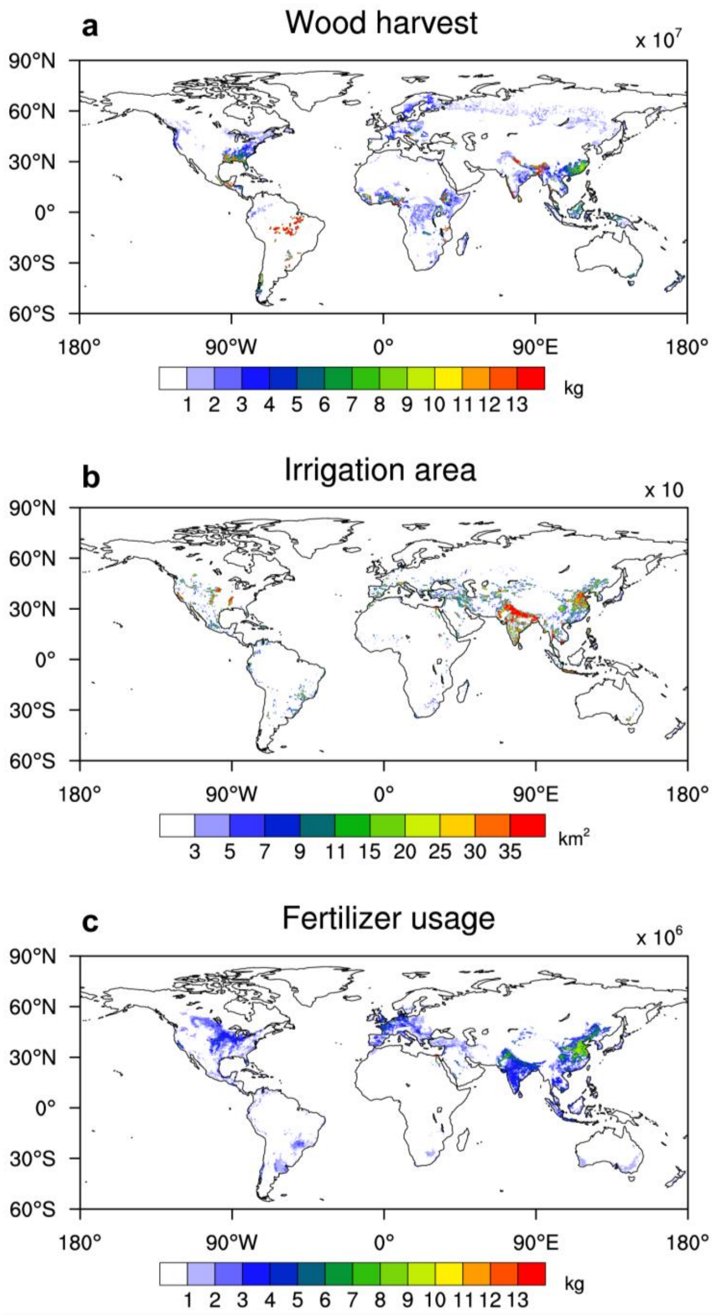

Supplementary Fig. 1 Spatial patterns of historical changes in land-use management from 1850 to 2014 (2014 minus 1850). **a** Wood harvest (10^7 kg). **b** Irrigated area (10 km^2). **c** Fertilizer usage (10^6 kg).

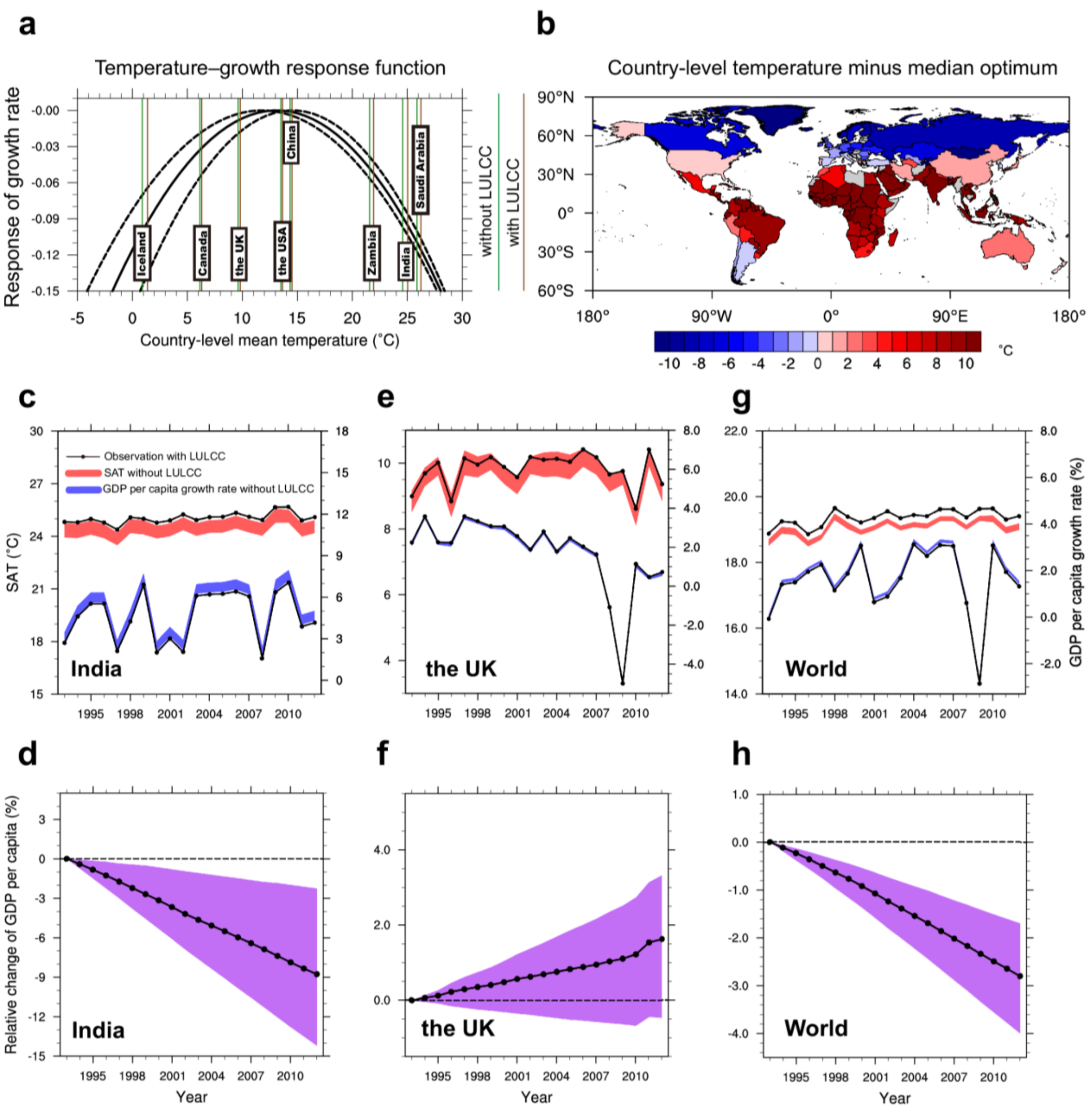

Supplementary Fig. 2 Country-level annual-mean surface air temperature (SAT) changes and associated economic impacts during 1993–2012 due to the biogeochemical (BGC) impact of historical land-use and land-cover change (LULCC). a Bootstrapped temperature–response function represented by 25th (left dashed curve), 50th (solid curve), and 75th (right dashed curve) percentiles of 1,000 members of the temperature optimum[1]. Vertical lines overlaid on the curves are annual mean temperatures from factual observations with LULCC (brown) and the counterfactual world without LULCC (green) for some representative countries. **b** Spatial pattern of the difference between the country-level annual mean temperature and median temperature

optimum. Countries and regions with missing values are shaded in grey. **c, e, g** The 25th–75th percentile range of SAT (red shading, in °C) and GDP per capita growth rate (blue shading, in %) in the counterfactual world without LULCC, both with corresponding observations in the factual world with LULCC (black dotted line) for (**c**) India, (**e**) the UK, and (**g**) the world. **d, f, h** Relative changes in GDP per capita (%) induced by LULCC for (**d**) India, (**f**) the UK, and (**h**) the world. The black dotted line and corresponding purple shading indicate the median and 25th–75th percentile range of ensemble members.

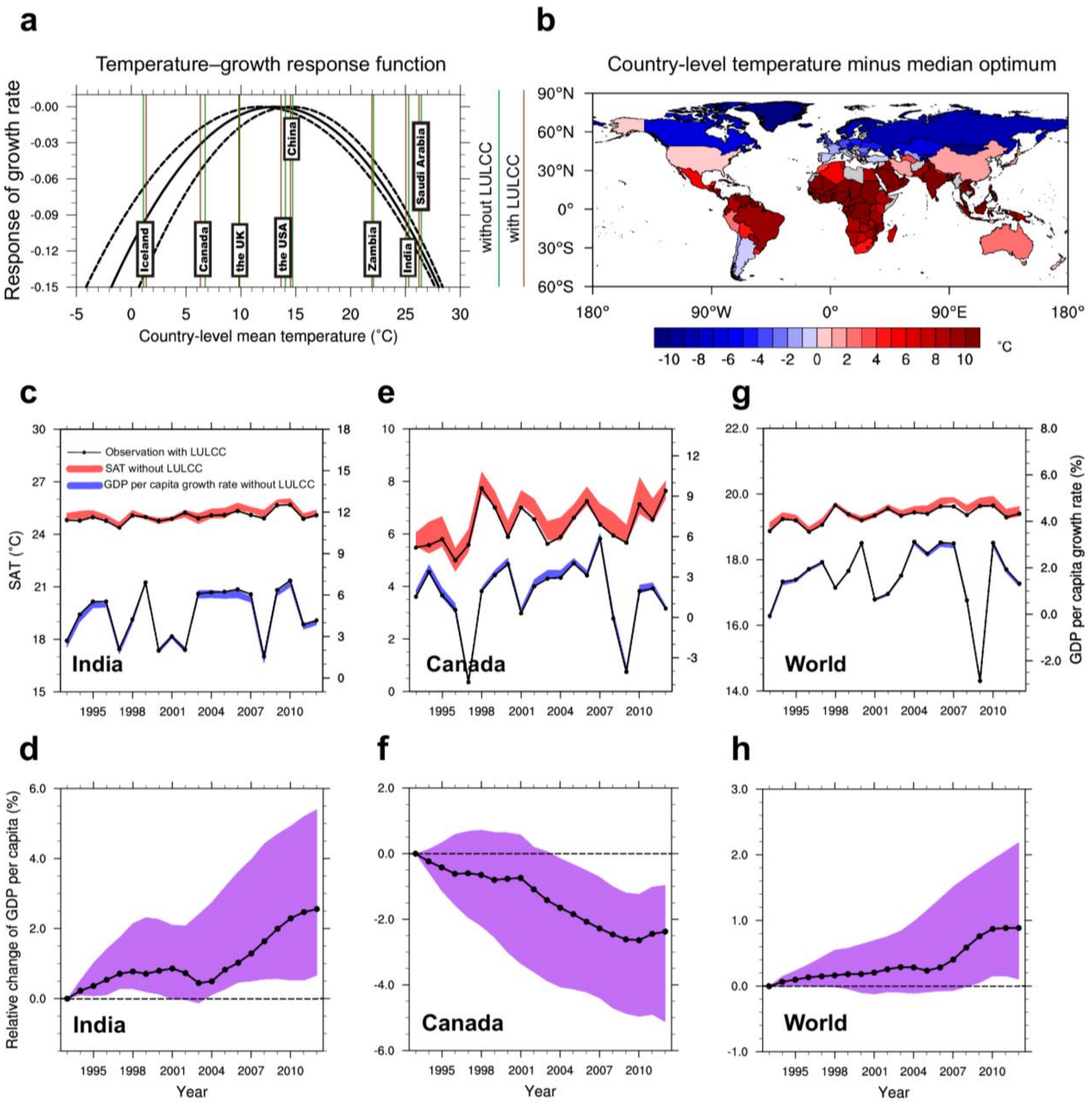

Supplementary Fig. 3 Same as Supplementary Fig. 2 but for the biogeophysical (BGP) impact of historical land-use and land-cover change (LULCC).

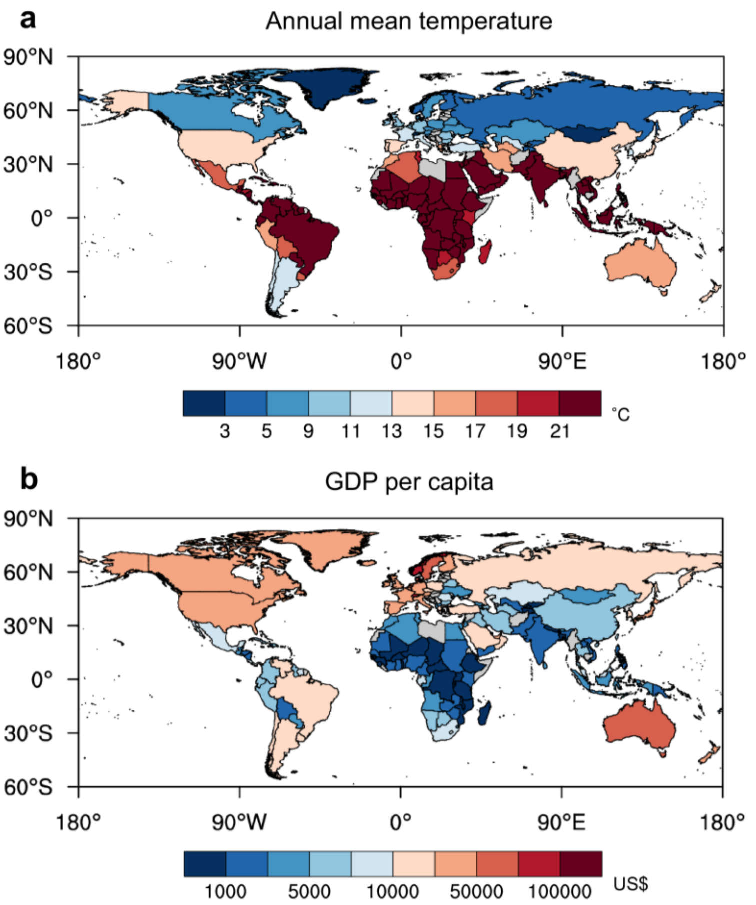

Supplementary Fig. 4 Spatial patterns of country-level annual mean temperature and GDP per capita. a Country-level annual mean temperature during 1993–2012 (in °C), weighted by population distribution in the country. **b** GDP per capita in 2012 (in $US\$_{2010}$). The blue/red color is split at the median temperature optimum (approximately 13°C) and global GDP per capita (approximately 10000 $US\$_{2010}$), respectively. Countries and regions not analyzed in our study are shaded in grey. Countries with a GDP per capita greater than 10000 $US\$_{2010}$ are grouped into economically advanced countries (in red). Otherwise, they are grouped into economically disadvantaged countries (in blue). Most economically disadvantaged countries are in the low latitudes with warm climates while economically advanced countries are generally situated in temperate and cool climates.

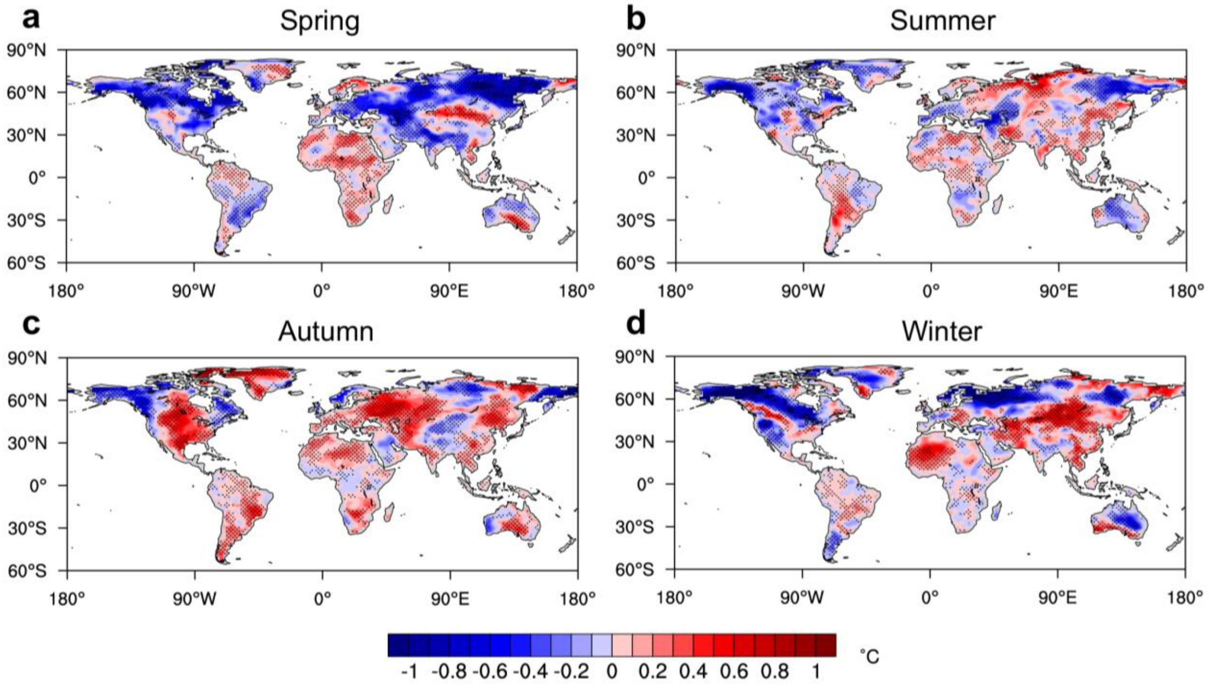

Supplementary Fig. 5 Spatial patterns of seasonal day-to-day surface air temperature (SAT) variability changes (°C) during 1993–2012 due to the combined impacts of historical land-use and land-cover change (LULCC). For boreal (**a**) spring, (**b**) summer, (**c**) autumn, and (**d**) winter. Dots indicate where more than two-thirds of members agree on the sign of response.

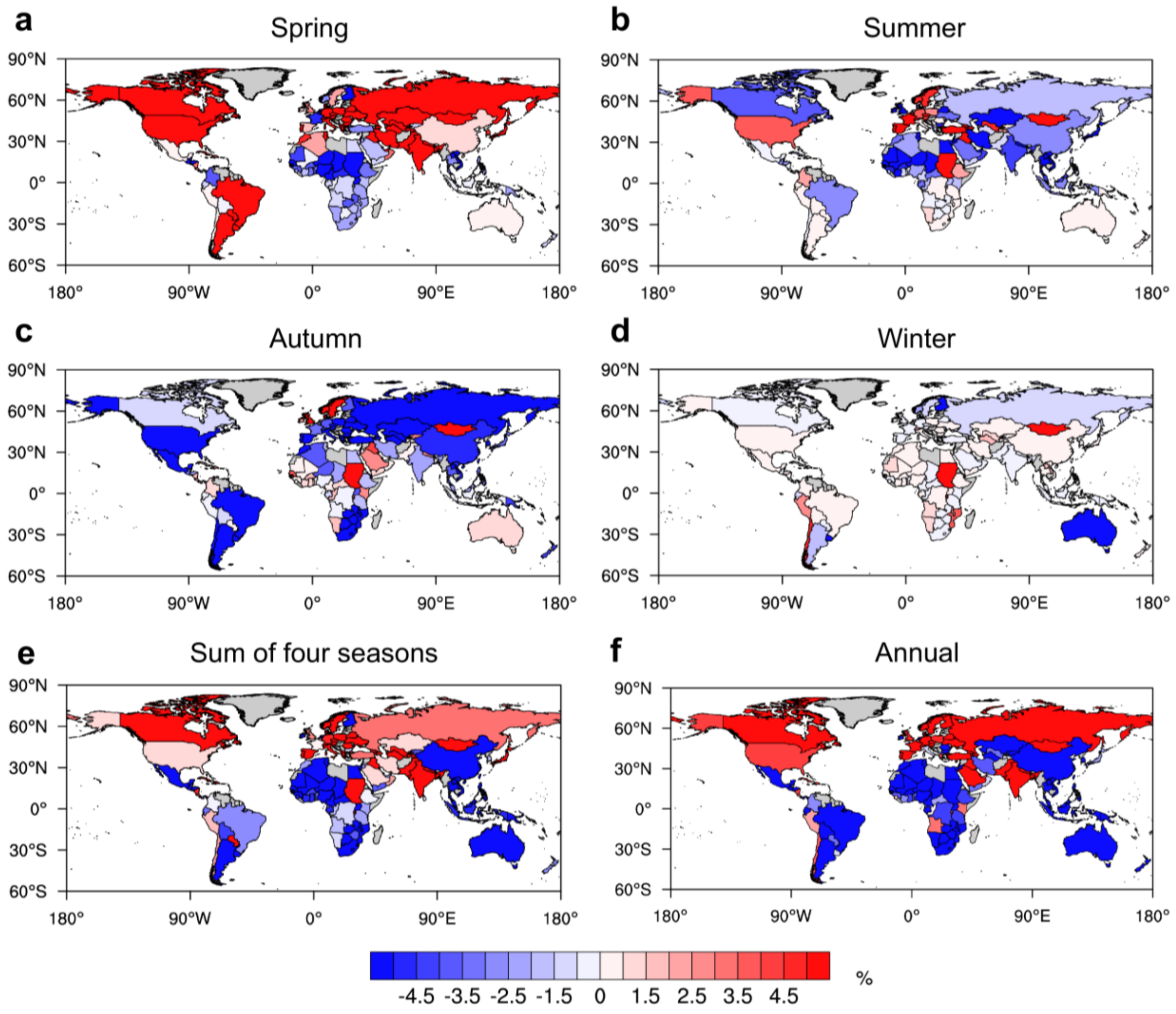

Supplementary Fig. 6 Country-level cumulative economic impacts via seasonal day-to-day surface air temperature (SAT) variability changes from 1993 to 2012 due to the combined impacts of historical land-use and land-cover change (LULCC). a–d Relative changes in GDP per capita (%) in 2012 due to day-to-day SAT variability changes in boreal (**a**) spring, (**b**) summer, (**c**) autumn, and (**d**) winter. **e** Total economic impacts of the four seasons. **f** The economic impact of annual mean day-to-day SAT variability changes (also shown in Fig. 8e). Countries and regions with missing values are shaded in grey.

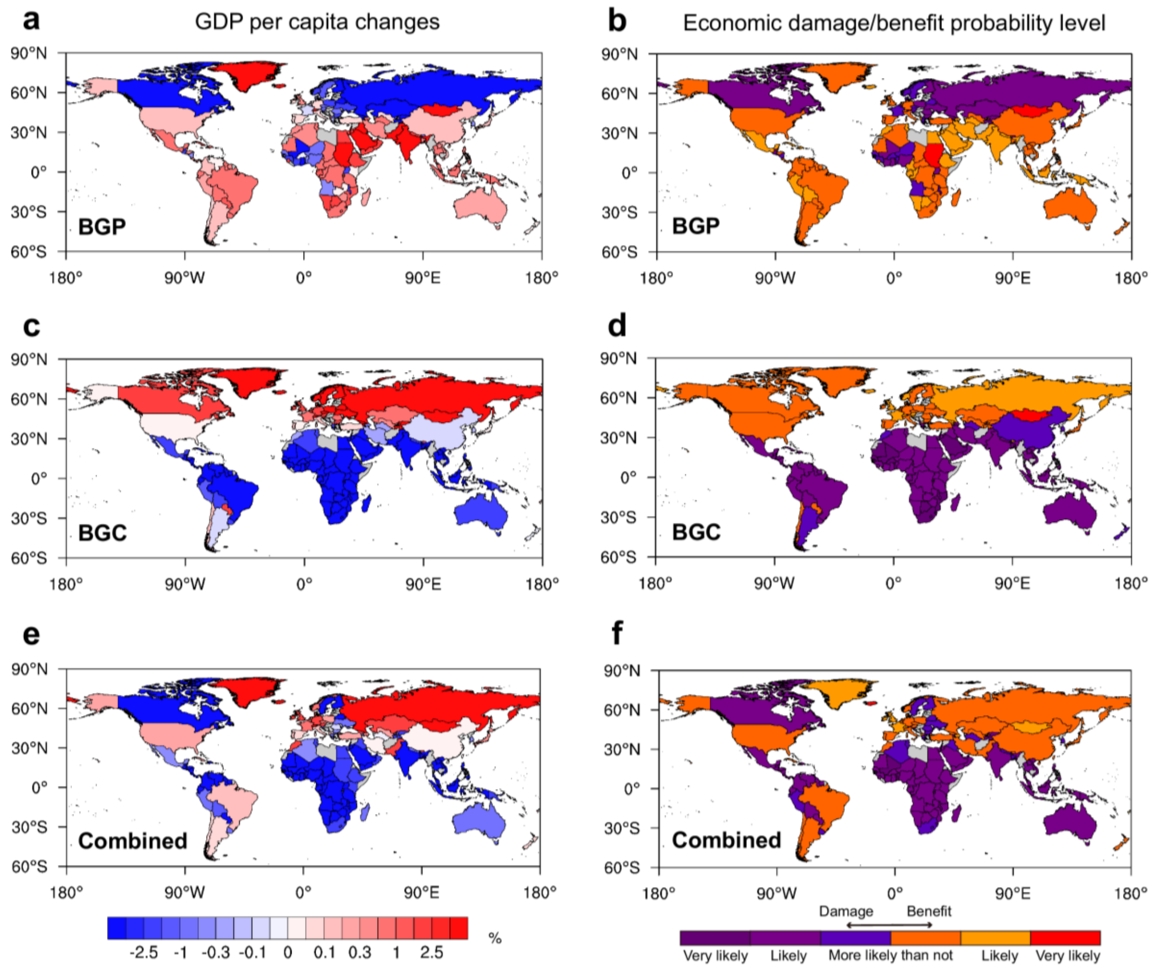

Supplementary Fig. 7 Country-level cumulative economic impacts via annual-mean surface air temperature (SAT) changes from 1993 to 2012 due to biogeophysical (BGP) and biogeochemical (BGC) impacts of historical land-use and land-cover change (LULCC) with the constraints of the MERRA2 reanalysis. a, c, e The ensemble median of the relative changes in GDP per capita (%) in 2012 induced by (**a**) BGP, (**c**) BGC, and (**e**) their combined impacts. **b, d, f** The corresponding probability level of the economic damage/benefit according to the IPCC uncertainty guidance[2] for (**b**) BGP, (**d**) BGC, and (**f**) their combined impacts. “Very likely”, “Likely”, and “More likely than not” indicate that more than 90%, two-thirds, and half of the members agree on the response, respectively. Countries and regions with missing values are shaded in grey.

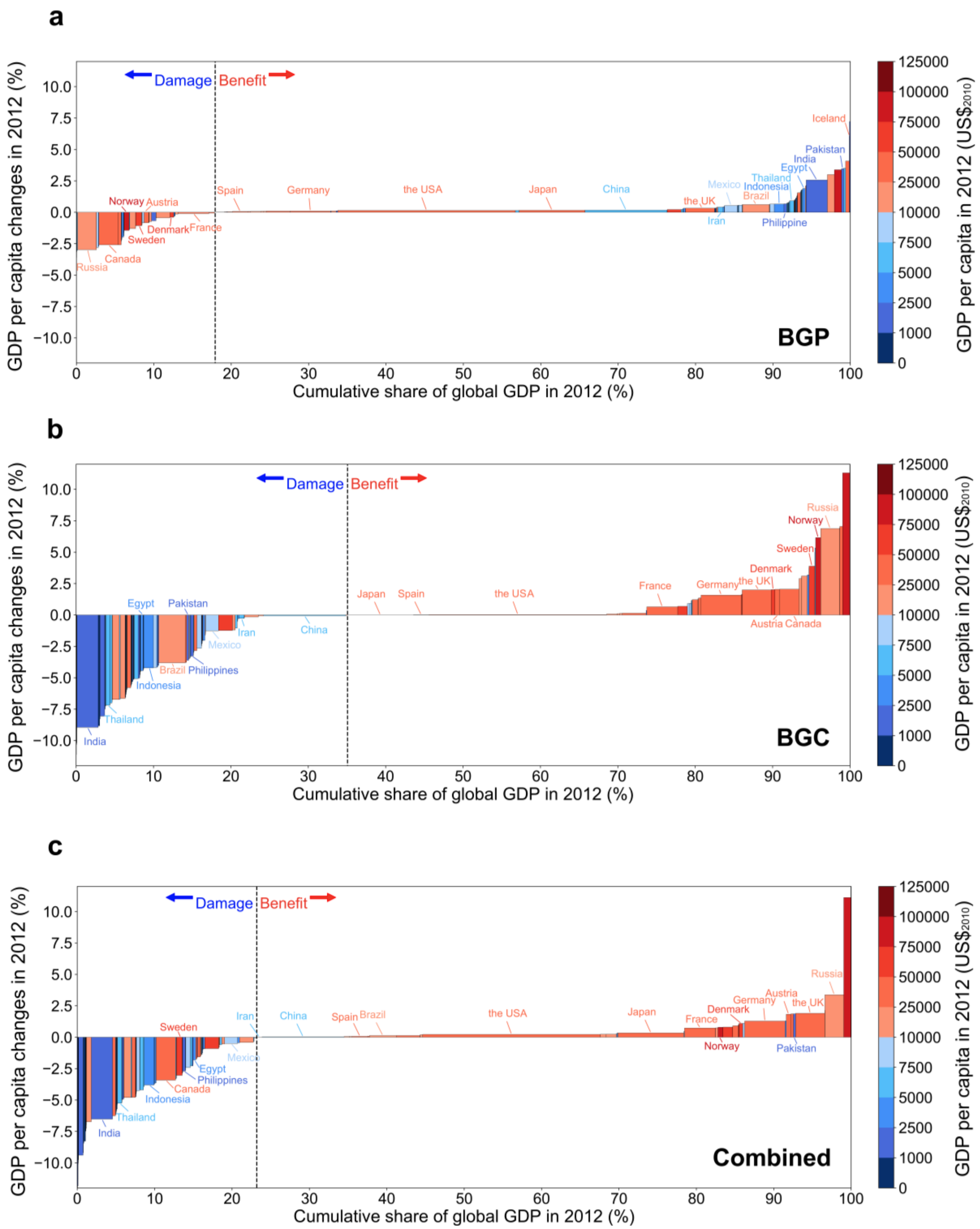

Supplementary Fig. 8 The biogeophysical (BGP) and biogeochemical (BGC) impacts of historical land-use and land-cover change (LULCC) on economies in countries of different economic conditions via annual-mean surface air temperature (SAT) changes with the constraints of the MERRA2 reanalysis. Countries are sorted by their relative changes in GDP per capita (%) in 2012 induced by (**a**) BGP, (**b**) BGC, and (**c**) their combined impacts, from

damages on the left to benefits on the right, with bars shaded by their corresponding GDP per capita in 2012 ($US\$_{2010}$). The blue/red color is split at the global GDP per capita in 2012 (approximately 10000 $US\$_{2010}$). Countries with a GDP per capita greater than 10000 $US\$_{2010}$ are grouped into economically advanced countries (in red). Otherwise, they are grouped into economically disadvantaged countries (in blue). The x-axis indicates the share of global GDP for each country.

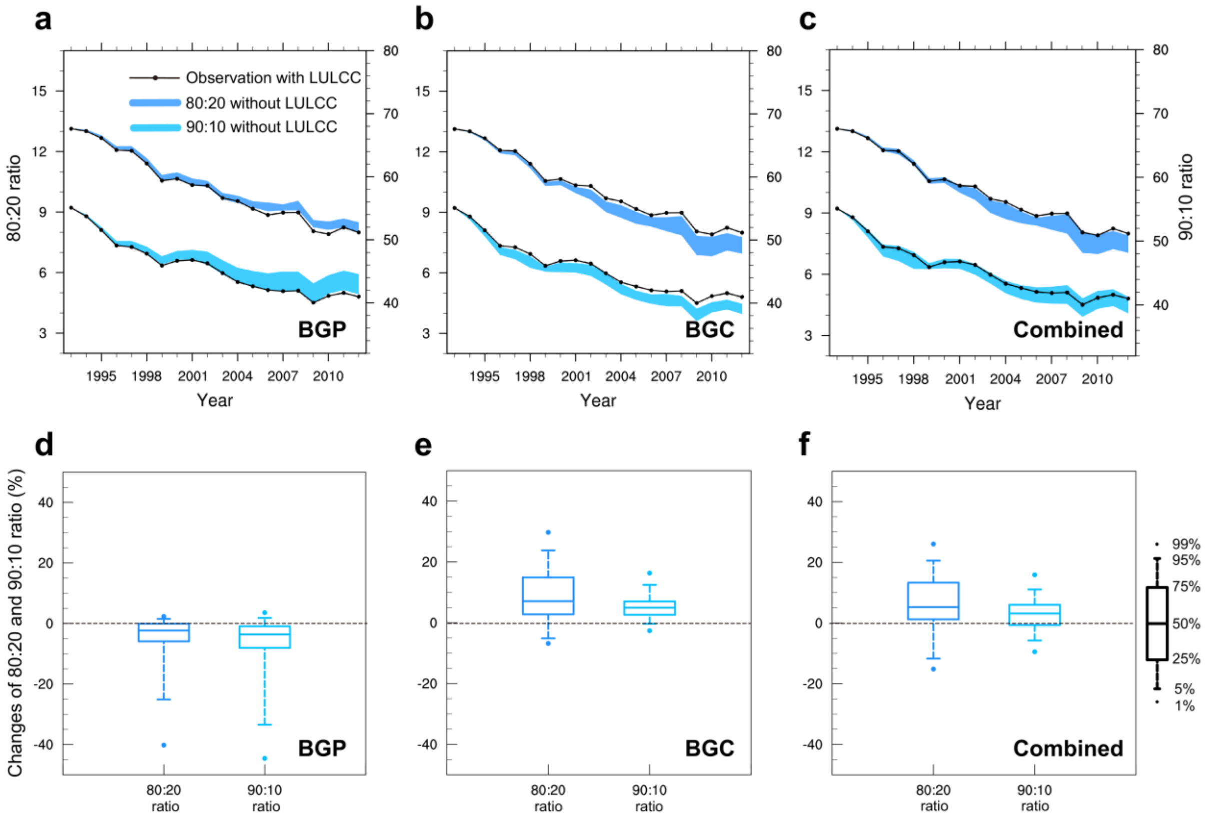

Supplementary Fig. 9 The biogeophysical (BGP) and biogeochemical (BGC) impacts of historical land-use and land-cover change (LULCC) on global economic inequality via annual mean surface air temperature (SAT) changes with the constraints of the MERRA2 reanalysis. a, b, c Time series of the 25th–75th percentile range of 80:20 (dark blue shading) and 90:10 (light blue shading) ratios of the population-weighted percentiles of GDP per capita in the counterfactual world without (**a**) BGP, (**b**) BGC, and (**c**) their combined impacts, both with corresponding observations in the factual world (black dotted line), from 1993 to 2012. **d, e, f** Relative changes (%) in 80:20 (dark blue box) and 90:10 (light blue box) ratios in 2012 induced by (**d**) BGP, (**e**) BGC, and (**f**) their combined impacts. The black box on the right shows the distribution of percentiles of box-and-whiskers. There are 16,000 members to estimate the BGP impact and 9,000 members to estimate the BGC impact and their combination.

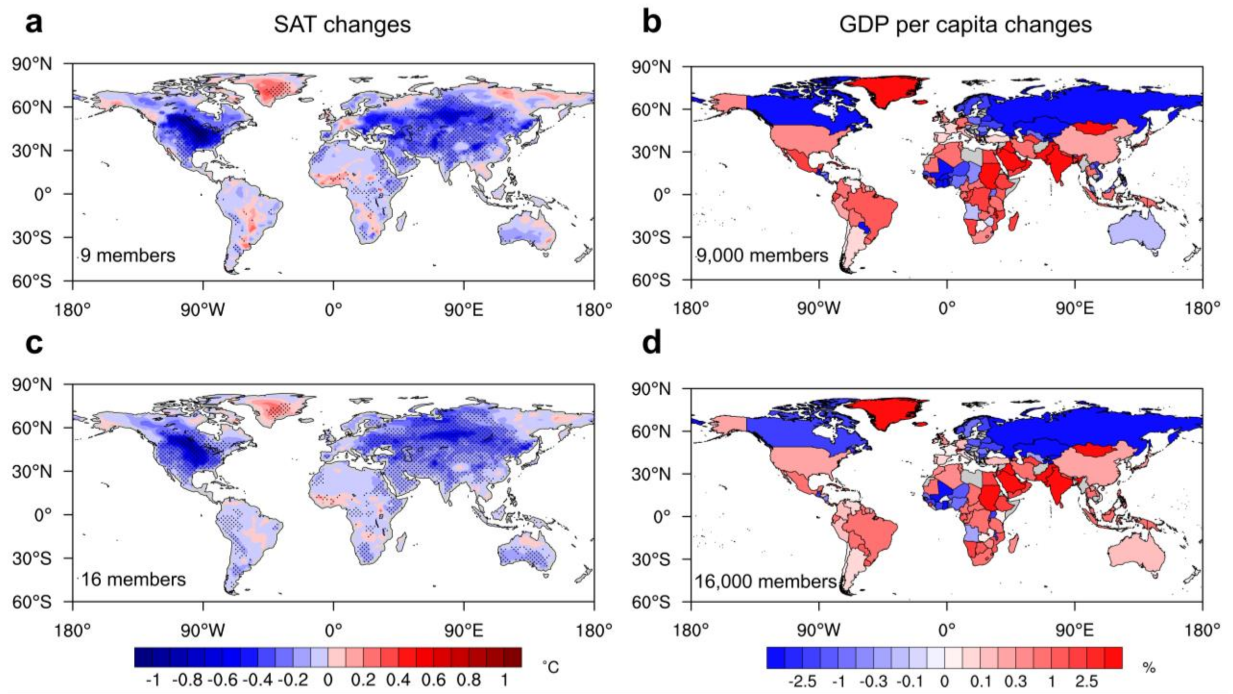

Supplementary Fig. 10 Global surface air temperature (SAT) and GDP per capita changes induced by the biogeophysical (BGP) impact of historical land-use and land-cover change (LULCC) for different ensemble sizes. a, c SAT changes (°C) for (**a**) 9 members and (**c**) 16 members. **b, d** GDP per capita changes (%) for (**b**) 9,000 members and (**d**) 16,000 members. The ensemble size is increased by 1,000 times for economic impacts because a country-level temperature change corresponds to 1,000 economic responses using the bootstrapped temperature–growth response function of 1,000 members.

Supplementary Table 1 The ensemble members and horizontal resolutions of CMIP6 models used for estimating individual and combined biogeophysical (BGP) and biogeochemical (BGC) impacts of historical land-use and land-cover change (LULCC) on annual mean surface air temperature (SAT) and day-to-day SAT variability.

Model	Annual-mean SAT			Day-to-day SAT variability	Resolution (longitude × latitude)
	BGP ensemble member	BGC ensemble member*	Combined ensemble member*	BGP, BGC & Combined ensemble member†	
BCC-CSM2-MR	1	–	–	–	1.1°×1.1°
CESM2	3	3	3	3	1.3°×0.9°
CMCC-ESM2	1	1	1	1	1.3 °×0.9°
EC-Earth3-Veg	1	1	1	–	0.7 °×0.7°
IPSL-CM6A-LR	4	–	–	–	2.5 °×1.3°
MPI-ESM1-2-LR	1	–	–	–	1.9 °×1.9°
NorESM2-LM	1	–	–	–	2.5 °×1.9°
UKESM1-0-LL	4	4	4	1	1.9 °×1.3°
Total	16	9	9	5	2.5 °×1.9°‡

* The ensemble member of the BGC impact is reduced due to many CMIP6 models without explicitly treating the carbon cycle processes in the "historical" and "hist-noLu" experiments. The ensemble member of the BGP impact is reduced to be consistent with the BGC impact when estimating their combined impacts.

† The ensemble member of the BGP and BGC impacts on day-to-day SAT variability is reduced due to many CMIP6 models without daily SAT outputs in the "hist-noLu" and "1pctCO2" experiments.

‡ To be consistent, all of the simulation outputs are interpolated to the same horizontal resolution of 2.5°×1.9°, the coarsest grid in the selected CMIP6 models.

Supplementary Table 2. Global economic impacts of annual mean surface air temperature (SAT) change induced by the biogeophysical (BGP) impact of historical land-use and land-cover change (LULCC), using various temperature–growth response functions.

Response functions	Schemes	Global GDP per capita changes (%)†	Probability of increased global GDP‡	80:20 ratio changes (%)§	Probability of decreased inequality	90:10 ratio changes (%)	Probability of decreased inequality
Burke et al[1]	country-lag0*	+0.88 (+0.10 to +2.19)	Likely	–2.30 (–5.75 to –0.06)	Likely	–3.11 (–7.14 to –0.77)	Likely
	country-lag1	+0.32 (–0.00 to +1.32)	Likely	–1.02 (–3.40 to +0.04)	Likely	–1.61 (–4.38 to –0.14)	Likely
	year	+0.92 (+0.10 to +2.25)	Likely	–2.30 (–5.71 to –0.05)	Likely	–3.08 (–7.10 to –0.77)	Likely
	year-blocks	+1.20 (+0.12 to +2.54)	Likely	–2.51 (–6.11 to –0.04)	Likely	–3.42 (–7.74 to –0.87)	Likely
Dell et al[3]	–	+0.45 (+0.05 to +0.86)	Likely	–2.86 (–5.89 to –0.71)	Likely	–4.42 (–9.48 to –1.39)	Likely
Pretis et al[4]	M1	+0.16 (–0.11 to +1.28)	More likely than not	–1.29 (–4.55 to –0.00)	Likely	–2.39 (–6.04 to +0.26)	Likely
	M2	+0.27 (–0.05 to +1.59)	Likely	–1.30 (–4.34 to +0.00)	Likely	–2.20 (–5.60 to –0.19)	Likely
	M3	+0.82 (+0.06 to +2.64)	Likely	–1.68 (–4.97 to +0.06)	Likely	–2.73 (–6.91 to –0.70)	Likely

* The temperature–growth response function is used for the central estimates in this study.

† The median and 25th–75th percentile range (in parentheses) of the 16,000 members of economic impacts.

‡ The probability levels of increased global GDP per capita and decreased global economic inequality according to the IPCC uncertainty guidance[2]. The levels of “Very likely”, “Likely”, and “More likely than not” indicate that more than 90%, two-thirds, and half of the members agree on the economic response, respectively.

§ Changes in the 80:20 and 90:10 ratios of the population-weighted percentile of GDP per capita (reflecting global economic inequality) relative to the counterfactual world without the BGP impact of LULCC.

Supplementary Table 3. Same as Supplementary Table 2, but for the biogeochemical (BGC) impact of historical land-use and land-cover change (LULCC).

Response functions	Schemes	Global GDP per capita changes (%)†	Probability of decreased global GDP‡	80:20 ratio changes (%)§	Probability of increased inequality	90:10 ratio changes (%)	Probability of increased inequality
Burke et al[1]	country-lag0*	–2.80 (–3.99 to –1.70)	Likely	+6.82 (+2.57 to +14.10)	Likely	+4.19 (+2.29 to +5.98)	Very likely
	country-lag1	–1.54 (–2.70 to –0.28)	Likely	+2.72 (+0.47 to +6.95)	Likely	+2.26 (+0.49 to +3.98)	Likely
	year	–2.87 (–4.10 to –1.76)	Likely	+6.85 (+2.45 to +13.78)	Likely	+4.12 (+2.31 to +5.88)	Very likely
	year-blocks	–3.26 (–4.48 to –2.32)	Likely	+7.86 (+2.90 to +15.09)	Likely	+4.32 (+2.57 to +6.26)	Very likely
Dell et al[3]	–	–1.17 (–1.66 to –0.79)	Likely	+11.85 (+2.89 to +20.75)	Likely	+5.23 (+2.66 to +7.49)	Likely
Pretis et al[4]	M1	–1.03 (–3.10 to +0.49)	Likely	+5.97 (+1.69 to +13.27)	Likely	+4.56 (+0.80 to +6.39)	Likely
	M2	–1.62 (–3.51 to +0.08)	Likely	+4.71 (+1.27 to +10.90)	Likely	+3.60 (+1.24 to +5.05)	Likely
	M3	–3.26 (–5.12 to –1.38)	Likely	+6.28 (+1.43 to +12.78)	Likely	+3.46 (+1.58 to +5.25)	Likely

* The temperature–growth response function is used for the central estimates in this study.

† The median and 25th–75th percentile range (in parentheses) of the 9,000 members of economic impacts.

‡ The probability levels of decreased global GDP per capita and increased global economic inequality according to the IPCC uncertainty guidance[2]. The levels of "Very likely", "Likely", and "More likely than not" indicate that more than 90%, two-thirds, and half of the members agree on the economic response, respectively.

§ Changes in the 80:20 and 90:10 ratios of the population-weighted percentile of GDP per capita (reflecting global economic inequality) relative to the counterfactual world without the BGC impact of LULCC.

Supplementary Table 4. Same as Supplementary Table 2, but for the combined biogeophysical (BGP) and biogeochemical (BGC) impacts of historical land-use and land-cover change (LULCC).

Response functions	Schemes	Global GDP changes (%)[†]	Probability of decreased global GDP[‡]	80:20 ratio changes (%)[§]	Probability of increased inequality	90:10 ratio changes (%)	Probability of increased inequality
Burke et al[1]	country-lag0[*]	–1.30 (–3.02 to –0.11)	Likely	+5.10 (+1.18 to +12.75)	Likely	+2.64 (–0.80 to +5.23)	likely
	country-lag1	–0.28 (–1.89 to +0.10)	Likely	+1.94 (+0.48 to +6.44)	Likely	+0.40 (–1.84 to +2.64)	More likely than not
	year	–1.36 (–3.10 to –0.12)	Likely	+5.15 (+1.18 to +12.66)	Likely	+2.48 (–0.78 to +5.01)	Likely
	year-blocks	–2.00 (–3.44 to –0.18)	Likely	+6.09 (+1.29 to +13.93)	Likely	+2.77 (–0.56 to +5.42)	Likely
Dell et al[3]	–	–0.75 (–1.42 to –0.21)	Likely	+8.49 (+0.90 to +17.45)	Likely	+1.52 (–1.92 to +5.41)	Likely
Pretis et al[4]	M1	–0.19 (–1.75 to +0.43)	More likely than not	+4.23 (+1.03 to +10.44)	Likely	+3.29 (–0.64 to +6.80)	Likely
	M2	–0.29 (–2.22 to +0.26)	Likely	+3.42 (+0.90 to +9.37)	Likely	+2.15 (–1.48 to +4.70)	Likely
	M3	–0.83 (–3.72 to +0.02)	Likely	+4.80 (+1.23 to +12.48)	Likely	+1.77 (–2.02 to +4.82)	More likely than not

[*] The temperature–growth response function is used for the central estimates in this study.

[†] The median and 25th–75th percentile range (in parentheses) of the 9,000 members of economic impacts.

‡ The probability levels of decreased global GDP per capita and increased global economic inequality according to the IPCC uncertainty guidance[2]. The levels of “Very likely”, “Likely”, and “More likely than not” indicate that more than 90%, two-thirds, and half of the members agree on the economic response, respectively.

§ Changes in the 80:20 and 90:10 ratios of the population-weighted percentile of GDP per capita (reflecting global economic inequality) relative to the counterfactual world without the combined BGP and BGC impacts of LULCC.

Supplementary References

1. Burke, M., Davis, W. M. & Diffenbaugh, N. S. Large potential reduction in economic damages under UN mitigation targets. *Nature* **557**, 549–553 (2018).
2. Mastrandrea, M. D. et al. The IPCC AR5 guidance note on consistent treatment of uncertainties: a common approach across the working groups. *Climatic Change* **108**, 675 (2011).
3. Dell, M., Jones, B. F. & Olken, B. A. Temperature Shocks and Economic Growth: Evidence from the Last Half Century. *Am Econ J Macroecon* **4**, 66–95 (2012).
4. Pretis, F., Schwarz, M., Tang, K., Haustein, K. & Allen, M. R. Uncertain impacts on economic growth when stabilizing global temperatures at 1.5°C or 2°C warming. *Philosophical Transactions Royal Soc Math Phys Eng Sci* **376**, 20160460 (2018).
